# Genome-Wide uH2A Localization Analysis Highlights Bmi1-Dependent Deposition of the Mark at Repressed Genes

**DOI:** 10.1371/journal.pgen.1000506

**Published:** 2009-06-05

**Authors:** Eric M. Kallin, Ru Cao, Raja Jothi, Kai Xia, Kairong Cui, Keji Zhao, Yi Zhang

**Affiliations:** 1 Howard Hughes Medical Institute, University of North Carolina at Chapel Hill, Chapel Hill, North Carolina, United States of America; 2 Department of Biochemistry and Biophysics, Lineberger Comprehensive Cancer Center, University of North Carolina at Chapel Hill, Chapel Hill, North Carolina, United States of America; 3 Biostatistics Branch, National Institute of Environmental Health Sciences, National Institutes of Health, Research Triangle Park, North Carolina, United States of America; 4 Laboratory of Molecular Immunology, The National Heart, Lung, and Blood Institute, National Institutes of Health, Bethesda, Maryland, United States of America; Friedrich Miescher Institute for Biomedical Research, Switzerland

## Abstract

Polycomb group (PcG) proteins control organism development by regulating the expression of developmental genes. Transcriptional regulation by PcG proteins is achieved, at least partly, through the PRC2-mediated methylation on lysine 27 of histone H3 (H3K27) and PRC1-mediated ubiquitylation on lysine 119 of histone H2A (uH2A). As an integral component of PRC1, Bmi1 has been demonstrated to be critical for H2A ubiquitylation. Although recent studies have revealed the genome-wide binding patterns of some of the PRC1 and PRC2 components, as well as the H3K27me3 mark, there have been no reports describing genome-wide localization of uH2A. Using the recently developed ChIP-Seq technology, here, we report genome-wide localization of the Bmi1-dependent uH2A mark in MEF cells. Gene promoter averaging analysis indicates a peak of uH2A just inside the transcription start site (TSS) of well-annotated genes. This peak is enriched at promoters containing the H3K27me3 mark and represents the least expressed genes in WT MEF cells. In addition, peak finding reveals regions of local uH2A enrichment throughout the mouse genome, including almost 700 gene promoters. Genes with promoter peaks of uH2A exhibit lower-level expression when compared to genes that do not contain promoter peaks of uH2A. Moreover, we demonstrate that genes with uH2A peaks have increased expression upon Bmi1 knockout. Importantly, local enrichment of uH2A is not limited to regions containing the H3K27me3 mark. We describe the enrichment of H2A ubiquitylation at high-density CpG promoters and provide evidence to suggest that DNA methylation may be linked to uH2A at these regions. Thus, our work not only reveals Bmi1-dependent H2A ubiquitylation, but also suggests that uH2A targeting in differentiated cells may employ a different mechanism from that in ES cells.

## Author Summary

A wealth of recent studies has demonstrated the role of Bmi1-stimulated histone ubiquitylation in the repression of transcription at targeted genetic loci. However, the repressive function of this mark has never been extrapolated genome-wide. We have used deep sequencing technology to explore the global deposition of Bmi1-dependent H2A ubiquitylation (uH2A) in mouse embryonic fibroblast cells. Our study confirms the gene-specific repressive function of the uH2A mark on a genome-wide scale. In addition, we also analyzed the general trends of uH2A distribution with respect to genomic elements, such as various classes of gene promoters and transcribed regions. Our work implies that the mechanism of uH2A distribution in differentiated cells may vary from that in embryonic stem cells. Given the importance of the uH2A modification in fundamental biological processes and cancer, insight into the distribution of this modification has reaching implications in understanding the contribution of epigenetic silencing to cellular physiology.

## Introduction

In higher eukaryotes, DNA is organized in the form of chromatin. The basic repeating unit of chromatin is called the nucleosome, which consists of 146 bp of DNA wrapped around a core histone octamer. One unique feature of core histones is their proclivity for covalent modification including acetylation, methylation, ubiquitylation, and phosphorylation [1]. In addition, DNA can be modified directly through methylation. These covalent modifications can affect gene transcription directly or indirectly through the recruitment of additional modulatory factors [2]. Therefore, different combinations of modifications on chromatin may ultimately determine distinct cellular states through regulating the transcriptional programs that cells adopt. Thus, identification and characterization of the proteins that are responsible for the placement and maintenance of these epigenetic marks is of great importance in understanding cellular proliferation and differentiation.

The addition of a single ubiquitin molecule to histone H2A at lysine 119 was first discovered over thirty years ago [3]. Classic experiments demonstrated that uH2A accounts for about 10% of total H2A [4]. Despite the knowledge of its existence, the identity of the responsible enzymes and the function of this modification have only recently begun to be elucidated. The first H2A ubiquitin E3 ligase was identified as the core components of the Polycomb repressive complex 1 (PRC1) composed of RING1/2, BMI1, and HPH2 [5]. Biochemical and functional analysis of the PRC1 complex has revealed RING2/Ring1b as the catalytic subunit, which can be greatly stimulated by Bmi1 and Ring1a, as loss function on any of these two proteins resulted in drastic genome-wide reduction of uH2A [6],[7]. Genome-wide location studies revealed that PRC1 occupies the promoters of a subset of Polycomb repressive complex 2 (PRC2) targets and both PRC1 and PRC2 are enriched at genes involved in developmental processes [8]–[10]. Recent studies have uncovered that Bmi1 homologs, such as Mel18 and NSPc1, can target the PRC1 complex in various cell types [11],[12]. In addition, a new E3 ligase for H2A, 2A-HUB, has also been reported [13] highlighting the fact that there must be Bmi1-dependent and Bmi1-independent pools of uH2A in the genome.

Unlike PRC1, PRC2 possesses H3K27-specific histone methyltransferase activity [14]. The discovery that a component of PRC1, Pc, can specifically recognize and bind to H3K27me3 [15]–[17] has prompted researchers to embrace a sequential recruitment model whereby PRC2-mediated H3K27 methylation contributes to PRC1 recruitment and subsequent ubiquitylation of histone H2A. This model is supported by three pieces of evidence. First, studies on Hox and *Ink4a/Arf* loci indicate that PRC1 knockdown reduced local uH2A levels which correlate with upregulation of gene expression [7],[18],[19]. Second, knockdown of the H3K27me3 demethylase, Utx, results in enrichment of both PRC1 and uH2A at PRC2 target genes [20]. Third, the majority of genome-wide Ring1b enriched regions in mouse embryonic stem cells (mES) co-localize with peaks of H3K27me3 [21].

In addition to the relationship between H3K27 methylation and H2A ubiquitylation, several studies also suggest a potential link between H3K27 methylation and DNA methylation. For example, H3K27 methylation has been demonstrated to play an important role in imprinted gene silencing [22],[23]. Components of PRC2, such as Ezh2, have been reported to interact with Dnmt1/3a/3b and are required for efficient DNA methylation at several target genes [24]. On the other hand, Dnmt1 may contribute to the recruitment of PRC1 as knockdown of Dnmt1 abrogates localization of PRC1 components to Polycomb bodies in cultured cells [25]. Consistent with this notion, recent studies have demonstrated that components of PRC1 can interact with a methyl-DNA binding protein, Mbd1 [26], and the Dnmt1-associated protein, Dmap1 [27]. Despite these reports, a general correlation between H3K27 methylation and DNA methylation may not exist as genome-wide epigenetic profiling revealed only a small subset of H3K27me3 positive promoters were found to be hypermethylated [28],[29]. Whether there exists a genome-wide link between PRC1 mediated H2A ubiquitylation and DNA methylation remains to be determined.

The advent of chromatin immunoprecipitation coupled to genomic tiling arrays (ChIP-chip) has provided scores of reports highlighting genome-wide maps of histone modifications [30], histone modifying enzyme binding profiles [9], and transcription modulators [31],[32]. Recent advances in ChIP-coupled deep sequencing have greatly expedited the tedious task of dissecting the interplay between epigenetic modifications and complex transcriptional output [33]. Although genome-wide analysis of most epigenetic marks [34]–[36], as well as transcription factors [37], has been reported for various cell lines, uH2A distribution remains a mystery. In addition, the recent discovery that the majority of uH2A in the fly genome is placed by a complex containing the Bmi1 homolog but lacking Pc [38] calls the generality of the sequential recruitment model into question.

To understand how uH2A fits into the complex epigenetic architecture associated with mammalian chromatin, we describe the genome-wide profile of Bmi1-dependent uH2A by comparing the enrichment of this mark in Bmi1 wild-type and null MEF cells. This analysis provides evidence that while Bmi1 dependent uH2A is enriched at genes containing the H3K27me3 mark, it is not limited to these regions. In addition, analysis of genome-wide DNA methylation patterns reveals a link between uH2A and DNA methylation in high-density CpG promoters. Transcription profiling of wild-type MEF cells indicates that Bmi1-dependent uH2A is enriched at genes with low levels of *de novo* expression. Finally, genes containing the highest levels of Bmi1 dependent uH2A at their promoters are expressed higher upon Bmi1 loss of function than genes harboring low levels of uH2A. Thus, our study uncovers some previously unrecognized features of uH2A.

## Results

### Generating Genome-Wide uH2A Modification Maps

In an effort to understand the function of the Bmi1-dependent uH2A epigenetic mark, we performed chromatin immunoprecipitation (ChIP) experiments using the well characterized uH2A monoclonal antibody E6C5 [5], [7], [39]–[41] in wild-type MEFs. The precipitated DNA was subjected to deep sequencing using the Solexa sequencing technology. As a control for non-specific background, and to focus the research on the Bmi1-dependent proportion of genomic uH2A, parallel ChIPs were also performed in Bmi1 null MEF cells. A previous report has shown that these cells undergo drastic reductions in global H2A ubiquitylation [7]. After sequencing 25 bp DNA fragments, the data retrieved from both cell types were mapped to the *mm*8 build of the *Mus musculus* genome. DNA tags which did not uniquely map to the genome were discarded and the resulting tag libraries consisted of over 6 million and 8 million unique reads for the wild-type and Bmi1 null MEF cells, respectively. We next performed normalization for the total number of uniquely mapped reads, and generated a final Bmi1-dependent uH2A summary file by aligning reads from both libraries and subtracting uH2A tags derived from Bmi1 null MEFs from those derived from wild-type MEFs. Density maps were created by ignoring negative tag density regions. UCSC genome browser tracks corresponding to the normalized uH2A in wild-type MEFs, Bmi1 null MEFs, and subtracted tag density plots are available from the NCBI GEO Database under accession number GSE15909.

### Enrichment of uH2A at the Majority of Gene Promoters Is Dependent on Bmi1 Function

**Figure 1:**
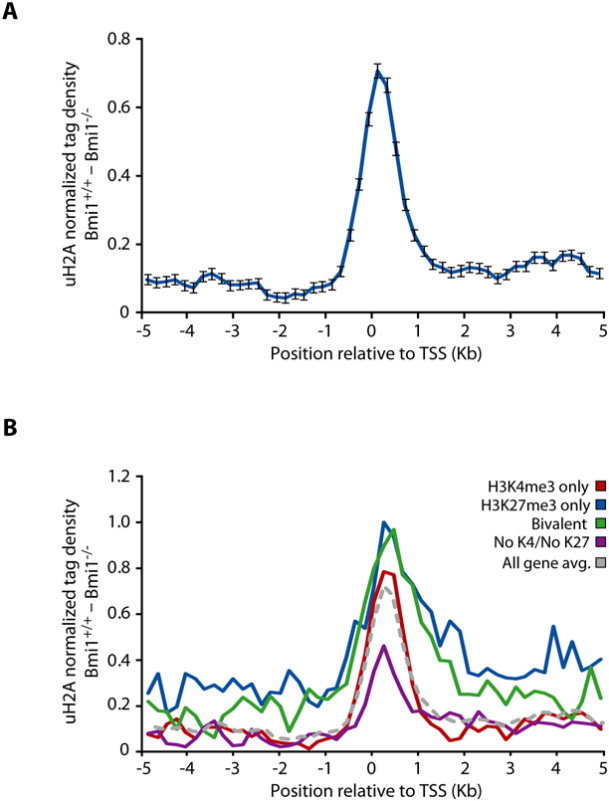
Bmi1-dependent promoter uH2A enrichment correlates with promoter H3K27me3 enrichment. (A) Profiles of per base pair Bmi1-dependent uH2A enrichment across the TSS of well annotated genes. Error bars represent s.e.m. of per base pair average for all genes analyzed (*n* = 15867). (B) Well-annotated genes were grouped by epigenetic modification state and the average normalized uH2A tag density was determined for each group. No K4/No K27 (*n* = 4690), purple; both H3K4me3 and H3K27me3 (Bivalent) (*n* = 1377), green; H3K27me3 only (*n* = 888), blue; H3K4me3 only (*n* = 8912), red; all genes (*n* = 15867), grey.

Given the enrichment of many epigenetic marks at gene promoters, we began our analysis of Bmi1-dependent uH2A localization by examining the area directly surrounding the transcription start site of well annotated genes. To this end, the average per base pair normalized density of Bmi1-dependent uH2A was determined for a 10 kb region surrounding well annotated TSSs at a 200 bp resolution (analyzed gene list is available in Table S1). This genome-wide averaging analysis revealed enrichment of the uH2A mark that peaked just inside the TSS (Figure 1A). Given the link between uH2A and PRC2 [25], we next set out to determine if a quantitative relationship existed between Bmi1-dependent promoter uH2A enrichment and promoter H3K4me3 and/or H3K27me3 placement. To this end, well-annotated genes were grouped into four classes based on the presence of H3K4me3 and H3K27me3 within their promoters, as determined by a previous study [35]. The same averaging analysis was applied to these separate groups of genes which included promoters containing H3K4me3 only, H3K27me3 only, both H3K4me3 and H3K27me3 (bivalent), or lacking both modifications (no K4/no K27). This analysis revealed Bmi1-dependent enrichment of average uH2A tag density for both the bivalent and H3K27me3 modified gene classes (Figure 1B, compare grey line to green and blue lines). Enrichment manifested in an increase in averaged peak height, as well as an overall broadening of the uH2A peak further into the body of K27me3 marked genes. This result is consistent with a role for PRC2 in the recruitment of PRC1 and subsequent ubiquitylation of histone H2A [7],[42]. Interestingly, Bmi1-dependent uH2A was still present at H3K4me3 genes at a level comparable to the all gene average (Figure 1B, red line). Furthermore, genes lacking both H3K4me3 and H3K27me3 exhibited a depletion of uH2A tag density (Figure 1B, purple line). Together these gene region averaging results reveal an overall enrichment of Bmi1-dependent uH2A at gene promoters which is biased towards genes marked by H3K27me3.

### Bmi1-Dependent uH2A Are Enriched at Specific Genomic Regions

To complement the genome-wide averaging studies described above, the uH2A tag library of wild-type MEFs was subjected to peak enrichment analysis using the TIROE algorithm [43] with the uH2A tag library derived from Bmi1 null MEFs set as background. This data processing allowed us to identify the most enriched Bmi1-dependent peaks of uH2A throughout the genome. A complete list of peak coordinates mapped to the *mm8* build of the mouse genome (Table S2) as well as a UCSC genome browser track showing the Bmi1 null subtracted tag density at peak regions (Dataset S1) is available as supplementary material online.

The TIROE algorithm identified 16,406 peaks of Bmi1-dependent uH2A throughout the genome with enrichment *P* values of at least 1e-5. We began our analysis of peak distribution by roughly dividing the genome into genic and non-genic regions as determined by the presence of transcribed regions annotated in the REFSEQ database. Interestingly, while genic regions correspond to only about 47% of the genome, they harbored 52% of the identified peaks (Table 1). Further division of the genic regions of the mouse genome into promoter and transcribed regions revealed that peak number enrichment was present in both of these sub-groups with 671 peaks (4.1%) falling within gene promoters and the remaining 7415 peaks (47.3%) localizing elsewhere along the transcribed body of the gene (Table 1, Table S3). Distribution analysis of defined peaks along transcribed regions revealed an increased tendency of uH2A peak localization towards the transcription termination site of genes (Figure S1A). However, in agreement with tag library averaging data presented in Figure 1A, peaks that fell within the promoter of genes (defined as −1 kb to +1 kb around TSSs) exhibited an average tag density higher than those lying within gene bodies (Figure S1B). Taken together, these data indicate that peaks of Bmi1-dependent uH2A are enriched within the transcribed regions of well-annotated genes with smaller peaks clustered towards the transcription termination site and larger peaks specifically occupying gene promoters.

**Table 1:** Bmi1-dependent peak distribution throughout the mouse genome.

	Genome*	Peak		*P* value**
	BP coverage	Number	BP coverage	
**Gene & Promoter**	893514855 (47.4%)	8086 (49.3%)	7748448 (52.4%)	<2.2e-16
**Promoter**	39206000 (2.1%)	671 (4.1%)	755102 (5.1%)	<2.2e-16
**Transcribed Region**	854308855 (45.3%)	7415 (45.2%)	6993346 (47.3%)	<2.2e-16
**Non-genic**	1770940233 (52.6%)	8320 (50.7%)	7046343 (47.6%)	<2.2e-16
**Total**	1884453825 (100%)	16406 (100%)	14794791 (100%)	

*: Total base pairs refer to alignable portion of the genome.

**: Two-sided proportional test of Genome BP coverage as compared to Peak BP coverage.

**Figure 2:**
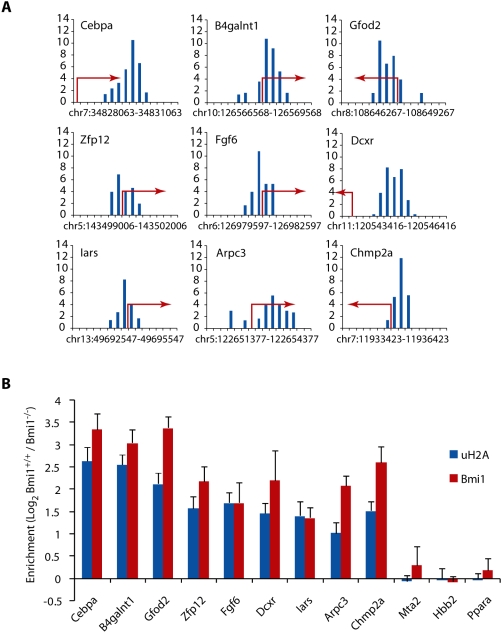
Bmi1-dependent promoter uH2A enrichment at a sub-set of gene promoters. (A) Bmi1-dependent tag density at selected gene promoters that contain peaks defined by the TIROE algorithm. Red arrow indicates the start and direction of transcription. (B) ChIP-qPCR validation of uH2A peaks (blue bars) and Bmi1 enrichment (red bars) was carried out in WT and Bmi1 null MEF cells. Enrichment was normalized to input control and data is presented as log_2_ value of WT versus Bmi1 null.

Given the enrichment of tag density at gene promoters as visualized by both genome-wide averaging (Figure 1A) and peak localization (Table 1 and Figure S1B), we next selected several representative TIROE peaks that localized within gene promoters to be verified by ChIP-qPCR. ChIP assays were carried out in both wild-type and Bmi1 null MEFs using antibodies against uH2A and Bmi1. The Bmi1 null subtracted tag density profiles surrounding the TSS of *Cebpa*, *B4galnt1*, *Gfod2*, *Zfp12*, *Fgf6*, *Dcxr*, *Iars*, *Arpc3* and *Chmp2a* are shown in Figure 2A. These peaks have TIROE enrichment *P* values of 2.4e-15, 1.2e-14, 1.3e-12, 7.0e-11, 9.2e-09, 1.1e-08, 1.1e-07, 2.2e-06, and 3.0e-06, respectively. ChIP assays not only confirmed the enrichment of uH2A at these loci in wild-type cells as compared to Bmi1 null cells, but also the presence of Bmi1 at these same regions in wild-type cells (Figure 2B). Similar enrichment of uH2A and Bmi1 localization was not detected within the promoters of *Mta2*, *Hbb2*, and *Ppara*; genes which do not contain peaks of the uH2A epigenetic mark (Figure 2B).

### Peaks of Bmi1-Dependent uH2A Overlap with Other Epigenetic Marks

Genetic studies have revealed a link between PRC1-catalyzed H2A ubiquitylation and PRC2-catalyzed H3K27 methylation. Biochemically, H3K27me3 has been shown to serve as a binding site for the recruitment of the PRC1 complex [16],[17]. These studies suggest that there should be a link between uH2A and H3K27me3. However, recent evidence from *Drosophila* has implicated another Bmi1 containing complex, dRAF, in H2A ubiquitylation. Interestingly, this complex lacks Pc and must utilize an alternative means of targeting [38]. To examine whether our dataset could shed light on the relationship between the two modifications, we investigated the overlap between peaks of Bmi1-dependent uH2A, H3K27me3, and H3K4me3. We reasoned that if an alternate method of E3 ligase recruitment was present in MEF cells then uH2A enrichment would be present in regions not marked by H3K27me3. Of the 4132 peaks of H3K27me3 identified in a previous study [35], about 15% (651) overlap with peaks of uH2A. Furthermore, about 11% of the 2,604 genomic regions defined as bivalent overlap with our uH2A dataset. Interestingly, about 5.6% of the 14,178 peaks of H3K4me3 exhibit co-localization with peaks of Bmi1-dependent uH2A. Together, these findings reveal that enriched regions of uH2A show a marked localization bias to genomic regions which also contain the H3K27me3 mark. However, they also indicate that the vast majority of Bmi1-dependent uH2A falls outside of regions containing this mark and suggests that an alternate method of ubiquitin E3 ligase recruitment may exist in MEF cells.

**Figure 3:**
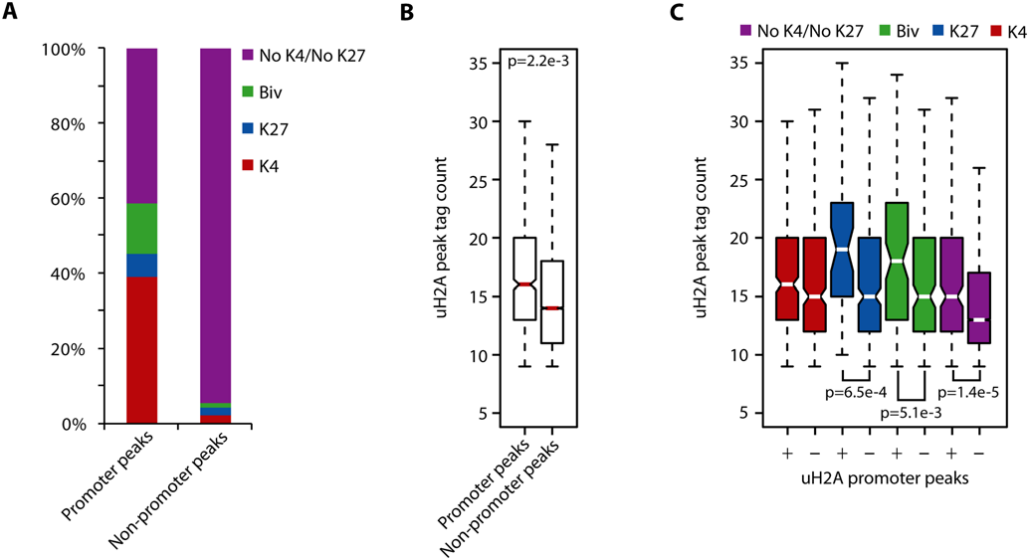
Promoter and non-promoter peaks of Bmi1-dependent uH2A are distinct. (A) Proportion of promoter uH2A peaks (*n* = 671) (left bar) and non-promoter uH2A peaks (*n* = 15735)(right bar) which overlap with H3K4/H3K27 methylation peaks. No K4/No K27, purple; both H3K4me3 and H3K27me3 (Bivalent), green; H3K27me3 only, blue; H3K4me3 only, red. (B) Box plot representation of peak tag density distribution for promoter and non-promoter uH2A peaks. Red lines indicate median values. *P* value derived from Wilcoxon signed-rank test. (C) Box plot representation of peak tag density distribution for promoter (+) and non-promoter (−) uH2A peaks further sub-divided by H3K4me3/H3K27me3 co-localization. No K4/No K27, purple; both H3K4me3 and H3K27me3 (Bivalent), green; H3K27me3 only, blue; H3K4me3 only, red. White lines indicate median values. *P* value derived from Wilcoxon signed-rank test.

Given that about half of all uH2A enriched regions in the genome fall outside of genic regions and only 4% are localized to gene promoters (Table 1, Table S3), we next explored possible differences between promoter and non-promoter peaks of uH2A with regards to H3K4me3 and H3K27me3 overlap. To this end, peaks were binned by their localization inside or outside of promoters, and the number of peaks from each of these groups which overlapped with additional epigenetic modifications was calculated. This analysis revealed that a higher proportion of promoter bound Bmi1-dependent uH2A peaks co-localized with either H3K4me3 or H3K27me3 when compared to peaks located outside of promoter regions (Figure 3A). This result is not surprising given the relative enrichment of H3K4/H3K27 within gene promoter regions. However, this finding reinforces the fact that Bmi1-dependent uH2A is distinct from the H3K27me3 mark with respect to the extent of its enrichment outside of genic regions.

We next investigated the local tag density of uH2A peaks both within and outside of promoters. Results presented in Figure 3B show that on average, peaks of uH2A localized within gene promoters are composed of significantly more tags than peaks lying outside of gene promoters (Wilcoxon *P* value = 2.2e-3). In addition, further sub-division of promoter and non-promoter peaks by H3K4me3 and H3K27me3 overlap reveals two additional pieces of information. First, promoter specific enrichment of uH2A peak tag density occurs at genes that also contain the H3K27me3 mark (Figure 3C, compare promoter peak data sets). This result is consistent with whole genome averaging analysis presented in Figure 1B. Second, there is a depletion of average peak tag density at non-promoter regions that do not contain either H3K4me3 or H3K27me3 (Fig 3C, compare non-promoter peak data sets). This finding, in conjunction with the fact that the vast majority of non-promoter uH2A falls outside of H3K4me3 and H3K27me3 regions (Figure 3A), suggests that the contribution of these independent peaks is driving overall depletion of tag density at non-promoter regions.

### Promoter-Bound uH2A Is Genetically Linked to DNA Methylation

**Figure 4:**
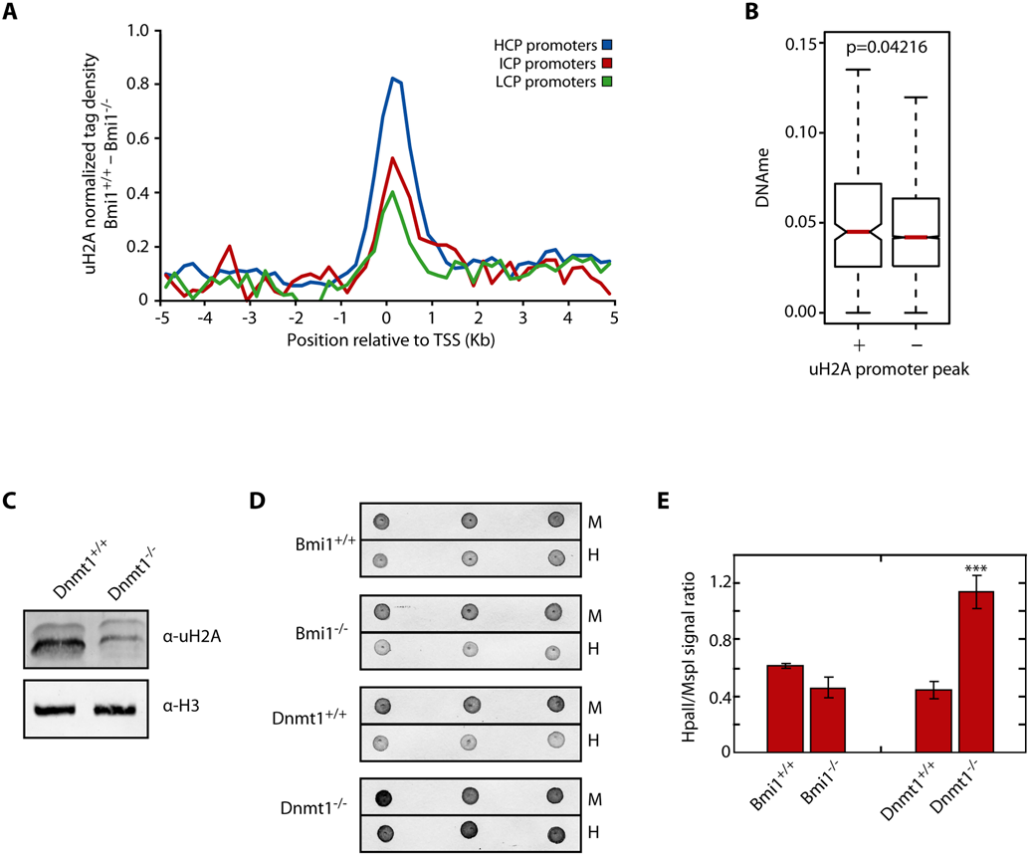
uH2A deposition is linked to DNA methylation. (A) Bmi1-dependent per base pair uH2A tag density was determined for genes defined as high CpG dinucleotide promoter content (HCP, blue) (*n* = 10,310), intermediate CpG dinucleotide promoter content (ICP, red) (*n* = 2889), low CpG dinucleotide promoter content (LCP, green) (*n* = 2668). (B) HCP class genes with available DNA methylation data were grouped based on the presence (+) (*n* = 266) or absence (−) (*n* = 8230) of a promoter bound peak of Bmi1-dependent uH2A and the distribution of DNA methylation values for each group was visualized using a standard box plot. Red lines indicate median values. *P* value derived from Wilcoxon signed-rank test. (C) Western blotting of uH2A was carried out using cell lysate prepared from wild-type and Dnmt1 null MEFs. H3 was used as a loading control. (D) Cytosine extension analysis was carried out using wild-type and Bmi1 null MEFs as well as Dnmt1 null and control cells. Biotin labeled restriction digests were spotted in triplicate onto a Nylon membrane and visualized using alkaline phosphatase. The procedure was repeated three independent times and data for one representative experiment is shown. (E) Data presented in panel D were quantified and presented as the ratio between methylation sensitive *HpaII* incorporation versus methylation insensitive *MspI* incorporation. Error bars represent s.e.m. (*n* = 3).

Previous studies indicate that PRC2 facilitates recruitment of DNA methyltransferases to at least a sub-set of Polycomb target genes [24]. This finding, together with the relationship between DNA methylation and transcriptional repression prompted us to investigate a possible link between genome-wide promoter uH2A and DNA methylation. To this end, we first analyzed uH2A localization in relation to CpG dinucleotide content surrounding the TSS of known genes. Following a previous characterization [35], we divided the genes into three groups defined as high level (*n* = 10,310), intermediate level (*n* = 2889), and low level (*n* = 2668) based on the density of CpGs within their promoter regions (HCP, ICP, and LCP, respectively) (Table S1). We repeated whole genome promoter averaging analysis and found that the promoter tag density of uH2A is strongly enriched at genes defined as HCP when compared to both ICP and LCP genes (Figure 4A). Recent reports have used bisulphite treatment coupled with deep sequencing to characterize the extent of DNA methylation at HCP group genes in ES, NPC, and MEFs [44]. Comparison of the available DNA methylation data with our promoter uH2A peaks revealed a correlation between DNA methylation levels and average uH2A tag density in the HCP group (Figure 4B). Specifically, HCP genes with promoter peaks of Bmi1-dependent uH2A had higher levels of average DNA methylation when compared to HCP genes without uH2A peaks (Wilcoxon *P* value = 0.0426).

To further characterize this correlation we asked whether the two modifications are genetically connected. Toward this end, we asked whether loss of DNA methylation would cause alteration in H2A ubiquitylation. A comparison of the uH2A levels in Dnmt1 null MEFs [45] revealed that loss of DNA methylation resulted in a significant decrease in uH2A levels (Figure 4C). To determine whether alteration in uH2A level affects DNA methylation, we compared the DNA methylation levels in wild-type and Bmi1 null MEFs by cytosine extension analysis. This technique takes advantage of DNA methylation sensitive/insensitive restriction enzymes and allows for the relative quantification of DNA methylation through end-labeling of genomic DNA digestion products [46]. Results shown in Figures 4D and 4E revealed a small, but statistically insignificant difference in the DNA methylation levels in the wild-type and Bmi1 null MEFs as determined by Student's t-test. As expected, parallel analysis revealed a drastic decrease of DNA methylation in the Dnmt1 null MEFs as indicated by the increased sensitivity of genomic DNA to the methylation sensitive restriction enzyme, *HpaII* (Figure 4D, E). Taken together, these data not only revealed a correlation between promoter bound uH2A and HCP promoter DNA methylation, but also provide evidence that DNA methylation may be upstream of H2A ubiquitylation.

### Bmi1-Dependent Promoter uH2A Marks Repressed Genes through Its Co-Localization with the H3K27me3 Mark

**Figure 5:**
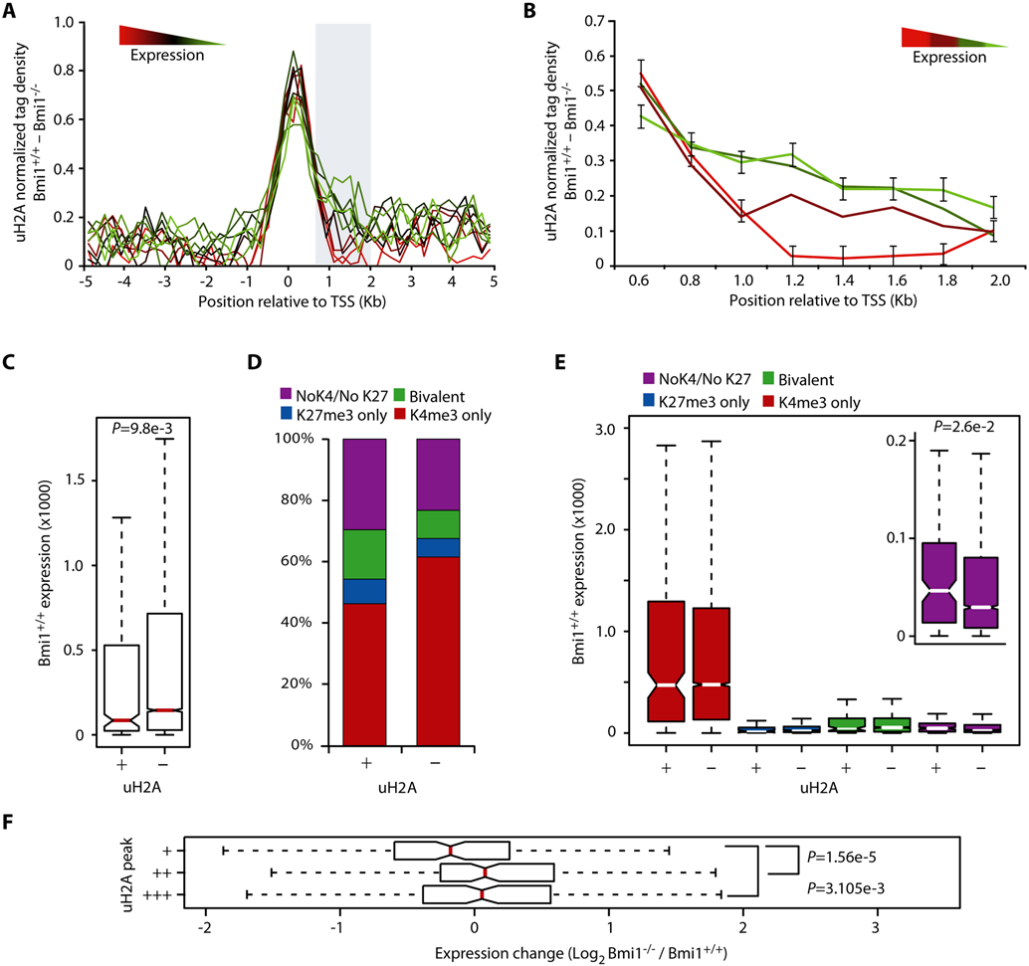
Bmi1-dependent uH2A is enriched at repressed genes. (A) Bmi1-dependent uH2A tag density surrounding the TSSs of genes was calculated and grouped into 10 bins (*n*≈1335 for each group) based on *de novo* gene expression levels in MEF cells. (B) Bmi1-dependent uH2A tag density was calculated for the region indicated in (A) for genes grouped based on *de novo* expression in MEF cells (*n* = 4007, *n* = 2674, *n* = 2668, *n* = 4005; highest expressed to lowest expressed). Error bars represent the s.e.m. of the highest expressed and lowest expressed gene groups. (C) WT MEF expression of genes with (+, *n* = 483) and without (−, *n* = 12,871) a peak of Bmi1-dependent uH2A was subjected to data distribution analysis by standard box plot. Red lines indicate median values. *P* value derived from Wilcoxon signed-rank test is indicated. (D) Gene promoters with (+) and without (−) peaks of uH2A were analyzed for the presence of H3K4/H3K27 methylation peaks. No K4/No K27, purple; both H3K4me3 and H3K27me3 (Bivalent), green; H3K27me3 only, blue; H3K4me3 only, red. (E) Box plot of expression data distribution for promoters with (+) and without (−) uH2A peaks in WT MEFs were further sub-divided by H3K4/H3K27 co-localization. No K4/No K27, purple; both H3K4me3 and H3K27me3 (Bivalent), green; H3K27me3 only, blue; H3K4me3 only, red. White lines indicate median values. *P* value derived from Wilcoxon signed-rank test is shown. (F) Genes containing a peak of Bmi1-dependent uH2A were divided into three groups based on increasing peak tag density (*n* = 157, 157, and 158, respectively) and the distribution of log_2_ expression change (Bmi1 null versus WT MEF cells) was presented by box plot. Red lines indicate median values. *P* value derived from Wilcoxon signed-rank test is shown.

Recent studies suggest a role for uH2A in the repression of developmental genes [7],[9],[47]. In addition, studies utilizing *in vitro* assembled chromatin templates have implicated uH2A in the repression of transcription initiation [48]. To determine whether Bmi1-dependent uH2A is a general indicator of transcriptional activity, we asked whether uH2A levels and gene expression levels have a general correlation. To this end, we profiled gene expression in wild-type MEFs using the Affymetrix Mouse Genome 430 2.0 microarray (Table S1) and analyzed the relationship between presence of uH2A peaks and gene activity for 13,354 genes. Based on their expression level, genes were grouped into 10 equal sized bins (Table S1) and were correlated with promoter uH2A enrichment levels using genome-wide tag density averaging. This analysis revealed that while the peak height of uH2A enrichment did not change much over these expression groups, the lowest expressed gene groups exhibited a broadening of the average uH2A tag density peak into the body of genes (Figure 5A). To better visualize this trend, the region spanning from +0.6 kb to +2.0 kb of genes was reanalyzed with genes divided into 4 groups. The resulting plot (Figure 5B) confirms Bmi1-dependent tag density broadening for genes present within the lowest expressed gene groups, a medium range broadening for genes in the third group, and no tag density broadening for the highest expressed genes present within the fourth group.

We next turned our attention to distinct promoter peaks of uH2A in an effort to understand gene specific outcomes as related to epigenetic mark deposition. In agreement with the data described above, expression level averaging of genes containing promoter peaks of uH2A revealed them to be significantly lower expressed then those without uH2A peaks (Wilcoxon *P* value = 9.8e-3) (Figure 5C). Taken together, these data indicate that an overall increase in Bmi1-dependent uH2A abundance can be found at silenced or low expressed genes; likewise, genes marked by promoter peaks of uH2A tend to be expressed at a lower level then genes lacking peaks of Bmi1-dependent uH2A.

The correlation between increased levels of uH2A and gene repression could be due to an intrinsic repressive effect of uH2A on transcription or due to an association of uH2A with other silencing epigenetic marks. To differentiate between these possibilities, we compared genes with promoter uH2A enrichment peaks to those without peaks in terms of their association with promoter H3K4me3 or H3K27me3 marks. This analysis revealed that a higher proportion of genes containing uH2A are also marked by H3K27me3 either alone or in the context of bivalent domains when compared with genes not marked by uH2A (Figure 5D). In addition, the uH2A positive gene set is enriched for promoters lacking both H3K27me3 and H3K4me3 as well as depleted for the H3K4me3 mark alone (Figure 5D). This indicates that grouping genes based on uH2A results in a population enriched for epigenetic marks tightly linked to gene repression and depletion of epigenetic marks tightly linked to gene expression. Given that this proportional shift could indicate that uH2A is passively correlated to gene repression, we next asked the question whether uH2A could function in conjunction with these additional epigenetic marks to fine-tune mRNA expression levels. To this end, we repeated the analysis described in Figure 5C for uH2A gene groups further sub-divided by additional promoter modifications and compared the average expression of these gene groups across classes (Figure 5E). Results confirm higher average expression of genes marked by H3K4me3 and drastically lower expression levels of genes containing promoter H3K27me3, H3K27me3 and H3K4me3 (bivalent), and neither mark (Figure 5E). Co-occurrence of uH2A with H3K4me3, H3K27me3, or bivalent domains did not result in any changes of average expression level when compared to genes harboring only the K4/K27 combinations. The sole significant change in WT expression level was a small increase for genes containing only the uH2A mark (Wilcoxon *P* value = 0.026) (Figure 5E). These results indicate that the correlation that exists between promoters bound by Bmi1-dependent uH2A and low level *de novo* gene expression is due to a proportional shift in other epigenetic marks that have a more profound effect on transcriptional state.

Even though Bmi1-dependent promoter uH2A is enriched at silenced genes, results of *de novo* expression analysis in wild-type cells were not able to ascertain an active function for uH2A in gene silencing. It is possible that a global role for uH2A in gene silencing was masked in this analysis by the presence of other epigenetic modifications which are more potent in enacting transcriptional control. To directly address a potential global role for Bmi1-dependent uH2A in gene silencing, we turned our attention to expression changes upon Bmi1 knock-out. To this end, we performed a microarray study on Bmi1 null MEFs and compared gene expression with that in the wild-type MEFs (Table S1). Of the 671 genes marked by promoter uH2A, we were able to generate reliable fold-change transcription data for 472 genes. We found that on average, this group was upregulated in Bmi1 null MEF cells by 1.5 fold. We next investigated whether the level of uH2A loss at genes in Bmi1 null cells was correlated to increased expression upon Bmi1 knock-out. For this analysis, genes containing Bmi1-dependent peaks of uH2A were sorted into three groups by increasing tag density (*n* = 157, 157, and 158, respectively), and both the average fold change as well as the data distribution of each group was determined. Interestingly, genes marked by the lowest levels of uH2A exhibited the smallest average increase in transcription upon Bmi1 knock-out when compared to both intermediate and high tag density groups (1.14, 1.98, and 1.37 fold, respectively). In addition, data distribution analysis and Wilcoxon signed-rank testing reveals that this finding is statistically significant (Figure 5F). Taken together, these data are consistent with a global role for Bmi1-dependent uH2A in gene silencing and extend gene specific analysis at important developmental regulators to genome-wide correlation.

## Discussion

Deep sequencing techniques have recently been used to map epigenetic marks in high definition throughout mammalian genomes [34],[35],[44]. Even though mono ubiquitylation of H2A was one of the first histone modifications identified [3], it remains among the least understood. Using a ChIP-Seq approach, here we analyzed the genome-wide distribution of Bmi1-dependent uH2A. This investigation revealed several interesting features of this epigenetic modification which serve as the basis for further studies.

### Bmi1-Dependent uH2A Distribution throughout Mammalian Chromatin

By combining genome averaging and peak localization analyses, this study reveals the first picture of the genome-wide localization of the uH2A mark and identifies Bmi1-dependent enrichment within both genic and non-genic regions of the mouse genome. On average, uH2A tag density is enriched at gene promoter regions (Figure 1A) with further enrichment at genes marked by H3K27me3 (Figure 1B). Peak analysis reveals that Bmi1-dependent uH2A enriched regions coincide with gene promoters at a much higher rate than can be expected by chance (Table 1), and these peaks encompass significantly higher tag values when compared with non-promoter peaks (Figure 3B). In addition, promoter peak tag values are enriched at genes also marked by H3K27me3 (Figure 3C). Interestingly, the gross distribution of genic uH2A peaks is skewed towards the 3′ end of genes, indicating that more regions of uH2A enrichment are found within these genic regions (Figure S1A). However, these peaks represent lower enrichment regions when compared to promoter peaks (Figure S1B). Together, these data support the notion that H3K27me3 can contribute to the recruitment of PRC2 and ubiquitylation of H2A in promoters [16],[17]. However, even though the highest regions of Bmi- dependent uH2A enrichment are at H3K27me3 genes, clear peaks also exist at genomic regions (both genic and non-genic) that do not contain this mark. This uH2A distribution pattern is very different from what would be expected given a recent study of Ring1b and Bmi1 binding profiles in mES cells [21] but are more consistent with a previous study which described a proportion of gene promoters positive for Ring1b/Bmi1 but negative for PRC2 binding [8]. Although this discrepancy may reflect the differences of uH2A distribution in ES cells and MEF cells, it is possible that similar to the observations in *Drosophila* [38], a Pc independent mechanism of Ring1b/Bmi1 recruitment may exist in MEF cells. Along these lines, about half of all the most enriched Bmi1-dependent uH2A regions lie outside of both promoter and transcribed regions of the genome (Table 1). Interestingly, these regions do not overlap significantly with peaks of H3K27me3.

### Relationship between uH2A and DNA Methylation

Since the initial finding linking Polycomb silencing to DNA methylation [24], genome-wide studies have called the generality of this association into question as very little overlap exists between genes methylated at H3K27 and genes that contain high levels of CpG island methylation associated with their promoters [28],[29],[49]. Recently, several reports have demonstrated a role for PRC1 in recognizing methylated DNA at specific loci or heterochromatic regions [25] either through Bmi1 interaction with Dmap1 [27] or Ring1b interaction with Mbd1 [26]. However, whether these specific instances of convergence of silencing pathways are linked to global gene regulation has not been determined. Here we provide evidence to support a functional link between DNA methylation and histone ubiquitylation through Bmi1-dependent mechanisms in a group of high CpG-containing genes. Not only does the level of DNA methylation increase at HCP promoters marked by uH2A (Figure 4B), a result that may be explained by an increase in H3K27 methylation on these genes, but knock-out of Dnmt1, which results in the ablation of CpG methylation genome wide, causes a global decrease in the uH2A levels (Figure 4C). In contrast, Bmi1 knock-out results in only a small, statistically insignificant, increase in global DNA methylation (Figure 4D). Taken together, these data suggest that DNA methylation may be upstream of Bmi1-dependent H2A ubiquitylation. Future work will reveal how DNA methylation contributes to H2A ubiquitylation.

### The Relationship between uH2A and Gene Expression

Recent studies in a limited gene set indicate that uH2A is mostly linked to transcriptional repression [7],[40],[48],[50]. Consistent with these studies, our genome-wide analysis indicate that Bmi1-dependent uH2A exhibits a broad enrichment at the most repressed genes in the mouse genome (Figure 5A and 5B). Peak centered analysis of promoter uH2A confirms this result (Figure 5C) and reveals that this enrichment is most likely a consequence of over-representation of the H3K27me3 mark (Figure 5C, 5D, and 5E). These results indicate that unlike H3K27me3 [34],[35], uH2A by itself is not an accurate predictor of *de novo* expression levels and could serve to explain earlier studies which have reported the presence of uH2A at actively transcribed genes [51],[52]. Instead, our results suggest that uH2A plays a much more refined role in the control of gene expression. Analysis of expression changes in Bmi1 knockout MEFs revealed that, on average, genes marked by promoter peaks of uH2A are upregulated upon Bmi1 knockout. In addition, the level of enrichment of these promoter uH2A peaks, as indicated by sequence tag density, reveal a clear increase in average fold change when higher density peaks are compared with lower density peaks (Figure 5F). Thus, our work extends gene specific studies and confirms the existence of a genome-wide link between uH2A and gene silencing.

## Materials and Methods

### Cell Lines, Cell Culture, and Antibodies

Mouse embryonic fibroblast cells were isolated from Bmi1 null and Bmi1 wild-type littermates and immortalized by expression of the TBX2.pBabePURO construct [18]. Both cell lines were maintained in Dulbecco's modified Eagles Medium supplemented with 10% (v/v) fetal bovine serum and 1× Penicillin/Streptomycin. Antibodies employed in this study are as follows: α-uH2A (Millipore, 05-678), α-Bmi1 (Millipore, 05-637), and α-H3 (Abcam, 1791).

### Chromatin Immunoprecipitation Assays

ChIP assays were performed as previously described [7] with the following alterations. Chromatin was prepared from one 15 cm^2^ plate grown to 95% confluence. After nuclei isolation, the pellet was resusupended in Solution B (20 mM Hepes pH 7.9, 25% [v/v] glycerol, 0.5% [v/v] NP-40, 0.42 M NaCl, 1.5 mM MgCl_2_, 1 mM CaCl), subjected to sonication, and treated with 30 units of micrococcal nuclease for 15 minutes to ensure mononucleosome resolution. Immunoprecipitations were carried out using 15 µg of antibody. For qPCR detection, the percent of IP enrichment as compared to input was calculated for both WT and Bmi1 null ChIPs using SYBR GreenER (Invitrogen) and data is presented as the fold change in percent input of WT versus Bmi1 null cells. All detection primers are listed in Table S4.

### Solexa Sequencing

Immunoprecipitated DNA fragments were blunt-ended, ligated to Solexa adaptors and sequenced using the Illumina 1G Genome Analyzer as previously described [34].

### Sequence Mapping

The 25 bp sequenced reads were obtained and mapped to the mouse genome (*mm*8 assembly) using the Solexa Analysis Pipeline, as previously described [34]. This yielded a BED file containing a total of 6.1 and 8.0 million unique tags for Bmi1 wild-type, and Bmi1 null libraries, respectively. Summary files were then created for each library by counting the number of tags falling into 200 bp genomic bins. A Bmi1-dependent uH2A summary file was created by applying a scaling factor to the WT file to equalize total tag count and subtracting the Bmi1 null tag count from the WT tag count within each genomic bin. Raw and processed sequencing data are available from the NCBI Gene Expression Omnibus (GEO) under accession number GSE15909.

### Expression Microarray Experiments

7 µg of total RNA from both Bmi1 wild-type and null MEFs was used to synthesize cDNA. A custom cDNA kit from Life Technologies was used with a T7-(dT)_24_ primer for this reaction. Biotinylated cRNA was then generated from the cDNA reaction using the BioArray High Yield RNA Transcript Kit. The cRNA was then fragmented in fragmentation buffer (5× fragmentation buffer: 200 mM Tris-acetate, pH 8.1, 500 mM KOAc, 150 mM MgOAc) at 94°C for 35 minutes before the chip hybridization. 15 µg of fragmented cRNA was then added to a hybridization cocktail (0.05 µg µl^−1^ fragmented cRNA, 50 pM control oligonucleotide B2, *BioB*, *BioC*, *BioD*, and *cre* hybridization controls, 0.1 mg ml^−1^ herring sperm DNA, 0.5 mg ml^−1^ acetylated BSA, 100 mM MES, 1 M [Na^+^], 20 mM EDTA, 0.01% [v/v] Tween 20). 10 µg of cRNA was used for hybridization. Affymetrix Mouse Genome 430 2.0 Arrays were hybridized for 16 hours at 45°C in the GeneChip Hybridization Oven 640. The arrays were washed and stained with R-phycoerythrin streptavidin in the GeneChip Fluidics Station 400. After this, the arrays were scanned with the Hewlett Packard GeneArray Scanner. Affymetrix GeneChip Microarray Suite 5.0 software was used for washing, scanning, and basic analysis. Sample quality was assessed by examination of 3′ to 5′ intensity ratios of certain genes. Raw and processed microarray data are available from the NCBI Gene Expression Omnibus (GEO) under accession number GSE15909.

### Cytosine Extension

The assay was adapted from a previous report [46]. Briefly, 100 ng of genomic DNA was digested to completion using *MspI* and *HpaII* (Fermentas) and the result was subjected to single nucleotide extension in the presence of biotin-labeled dCTP (Invitrogen). One-fiftieth of the final reaction was manually spotted on (+) nylon membrane, incubated in 0.4N NaOH, neutralized with 1× TBS, baked at 80°C for 20 minutes, and blocked overnight at 65°C in blocking buffer (4× SSPE, 6× Denhardt's, 300 µg ml^−1^ salmon sperm DNA, 0.1% [w/v] SDS). The blot was incubated with a 1∶5000 dilution of streptavidin conjugated alkaline phosphatase (Pierce) in blocking buffer for 20 minutes at room temperature, washed 3×15 minutes in TBST, and visualized using BCIP/NBT solution (Sigma). The developed blot was scanned and signal was quantified using NIH ImageJ software (http://rsb.info.nih.gov/ij/).

### Data Analysis

#### Determination of Bmi1-dependent peaks of uH2A

Determination of Bmi1-dependent peaks of uH2A: Peak enrichment calls were generated by the modified TIROE algorithm. The original TIROE algorithm, as described in [43], does not use a control background data while identifying peaks in the test data. In the modified version, a “fold enrichment” score was calculated for each candidate peak by computing the ratio of the number of tags within that candidate peak in the test data to the number of tags within the genomic region corresponding to the peak in the background control data. The fold score is normalized by the total number of tags within the test and control sample. Only those peaks with fold score greater than or equal to a set threshold are reported as final peaks. In our case, the input was set to the mapped tag BED file corresponding to wild-type ChIP-Seq and with the Bmi1 null BED file set to background. Parameter cut-offs were set as follows: *P*-value (p)≤1e-5, Fold enrichment cutoff (f)≥5, and the average DNA fragment length (f) was estimated to be 232 from the reads in the input data using the algorithm provided in [53].

#### Gene analysis groupings

REFSEQ gene coordinates were extracted from the UCSC table browser and the list was parsed to only consider one isoform per annotated gene. Isoform selection was carried out by first searching for the 3′ most transcription start sites. If a single gene had more than one isoform sharing a single TSS, then the longest isoform was kept for analysis. Promoters were defined as −1 kb to +1 kb surrounding TSSs and previously published peak data sets [35],[44] composed of H3K4me3, H3K27me3, CpG nucleotide density calls, and HCP promoter methylation analyses were re-mapped onto this gene list. Table S1 lists all genes used in this analysis as well as promoter definitions and dataset overlap. High resolution mapping surrounding gene TSSs was accomplished by extracting per base pair Bmi1-dependent uH2A tag density reads at 200 bp intervals surrounding TSSs.

#### Peak distribution analysis

The location of TIROE uH2A peaks was carried out in relation to the alignable genome as previously defined [35].

#### Standard Error calculation

Whole genome averaging analysis error bars represent the standard error of the mean (s.e.m.) and *P* values were determined using student's *t* tests. Two-sided proportional testing, Wilcoxon signed-rank testing, and box plot generation were carried out using the R package.

## Supporting Information

Figure S1 Genic peak distribution analysis reveals peak number enrichment towards the transcription termination site of genes and peak tag density enrichment within gene promoters. (A) Distribution histogram of peak location along the transcribed region of well-annotated genes (TSS, transcription start site; TES, transcription end site). (B) Genic uH2A peaks were grouped by their location within transcribed genes and the tag density data distribution was visualized by standard box plot. Red lines indicate median values. *P* value derived from Wilcoxon signed-rank test. *, **, and *** respectively indicate *P* value of 2.2e-14, 1.1e-15, 2.6e-10.
(0.51 MB PDF)

Table S1 REFSEQ genes included in this analysis.
(3.82 MB XLS)

Table S2 Bmi1-dependent uH2A peak regions as determined by TIROE.
(1.67 MB XLS)

Table S3 Bmi1-dependent peaks corresponding to gene promoter regions.
(0.08 MB XLS)

Table S4 List of primers used in this study.
(0.01 MB PDF)

Dataset S1 UCSC genome browser track of summary tag counts throughout Bmi1-dependent uH2A peak regions.
(1.38 MB GZ)
