# Supplementary figures and images for: Genome-Wide uH2A Localization Analysis Highlights Bmi1-Dependent Deposition of the Mark at Repressed Genes
Source: PLoS Genet. 2009 Jun 5;5(6):e1000506. doi: 10.1371/journal.pgen.1000506 (PMC2683938; doi:10.1371/journal.pgen.1000506)

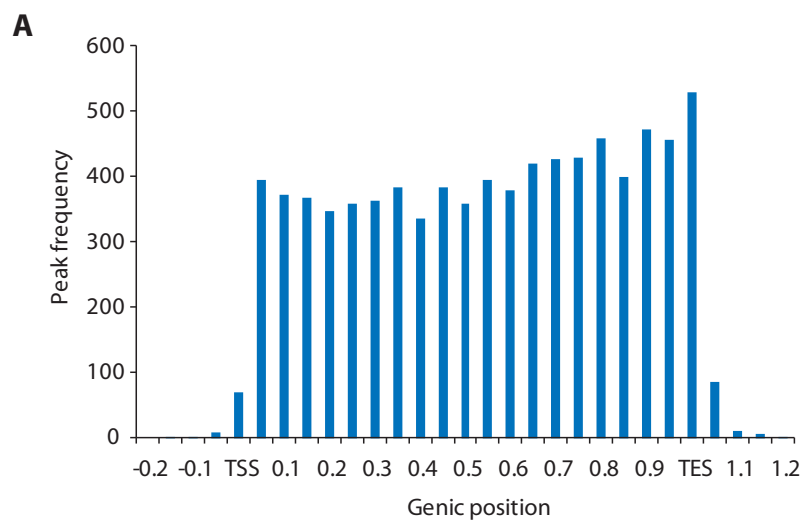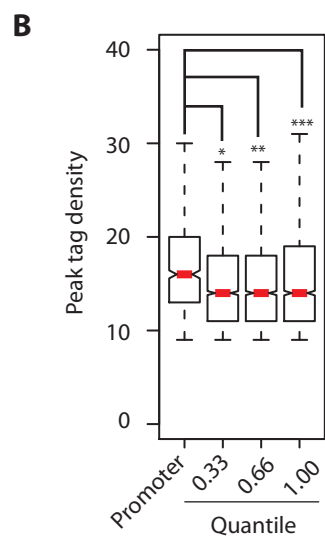

Supplement: Figure S1 — Genic peak distribution analysis reveals peak number enrichment towards the transcription termination site of genes and peak tag density enrichment within gene promoters. (A) Distribution histogram of peak location along the transcribed region of well-annotated genes (TSS, transcription start site; TES, transcription end site). (B) Genic uH2A peaks were grouped by their location within transcribed genes and the tag density data distribution was visualized by standard box plot. Red lines indicate median values. P value derived from Wilcoxon signed-rank test. *, **, and *** respectively indicate P value of 2.2e-14, 1.1e-15, 2.6e-10. (0.51 MB PDF) [file pgen.1000506.s001.pdf]
