# Supplementary material for: Genome-Wide uH2A Localization Analysis Highlights Bmi1-Dependent Deposition of the Mark at Repressed Genes
Source: PLoS Genet. 2009 Jun 5;5(6):e1000506. doi: 10.1371/journal.pgen.1000506 (PMC2683938; doi:10.1371/journal.pgen.1000506)
Supplement: Table S4 — List of primers used in this study. (0.01 MB PDF) [file pgen.1000506.s005.pdf]

Supplementary Table 4: ChIP-qPCR primers used in this study.

Gene	Forward Primer	Reverse Primer
Cebpa	CAG GGC AGG AGG AAG ATA CA	CAC CTA AGT CCC TCC CCT CT
B4galnt1	GGC GGA TTT ACG ATC CAG T	GAC TCC GGG GCT TTG TAG AC
Gfod2	GGA TGG GTT AGG TTC AAG CA	ACG GCA TTG AGT ACG AGG TC
Zfp12	GTG CGC ACT TCT GTT TGT GT	AAA GCT GCG AGC GTA GAG AC
Fgf6	CTG AAG CAG GCT TTG GTT TC	ACC GCC CTT CTT GTT TTT CT
Dcxr	GAG CGC ATA CTC CTC CAC TC	TGT TGG ACT TGA GGT GGT CA
Iars	GGA GCG TCT TCT CCT TCC TT	TGC CAT CCA ACA CCT ACA AA
Arpc3	ATT TCC CCC TTG TCA TTT CC	TCC ATC AGG GAA GTG AGG TC
Chmp2a	AAC CAA ATT AGG CCC ACA CA	GGT GCA AGC AAT GGA AGA AT
Mta2	TTC GTC TGG AGC TGA GCT TT	TCC CAC CCG GTA CAT GTT AG
Hbb2	GCCCAGGCTTAAGACATTTGAG	GAATTAGCTGCAAGGATAAGAACAGA
Ppara	GAGACCCTGGAGATGGTTGA	GTGTGCTTGTGTGCATGTGA
